# Understanding the Role of Intrinsic Disorder of Viral Proteins in the Oncogenicity of Different Types of HPV

**DOI:** 10.3390/ijms19010198

**Published:** 2018-01-09

**Authors:** Elvira Regina Tamarozzi, Silvana Giuliatti

**Affiliations:** Department of Genetics, School of Medicine of Ribeirão Preto, University of São Paulo, Sao Paulo 14049-900, Brazil; elvira@usp.br

**Keywords:** intrinsically disordered proteins, computational prediction, E6 protein, HPV, cancer

## Abstract

Intrinsic disorder is very important in the biological function of several proteins, and is directly linked to their foldability during interaction with their targets. There is a close relationship between the intrinsically disordered proteins and the process of carcinogenesis involving viral pathogens. Among these pathogens, we have highlighted the human papillomavirus (HPV) in this study. HPV is currently among the most common sexually transmitted infections, besides being the cause of several types of cancer. HPVs are divided into two groups, called high- and low-risk, based on their oncogenic potential. The high-risk HPV E6 protein has been the target of much research, in seeking treatments against HPV, due to its direct involvement in the process of cell cycle control. To understand the role of intrinsic disorder of the viral proteins in the oncogenic potential of different HPV types, the structural characteristics of intrinsically disordered regions of high and low-risk HPV E6 proteins were analyzed. In silico analyses of primary sequences, prediction of tertiary structures, and analyses of molecular dynamics allowed the observation of the behavior of such disordered regions in these proteins, thereby proving a direct relationship of structural variation with the degree of oncogenicity of HPVs. The results obtained may contribute to the development of new therapies, targeting the E6 oncoprotein, for the treatment of HPV-associated diseases.

## 1. Introduction

The function of proteins is not solely linked to their rigid three-dimensional structure, as was thought less than three decades ago. Studies with physiologically unstructured, yet biologically active, proteins were conducted to challenge the concept based on the structure-function paradigm, according to which the function of a protein is exclusively determined by its rigid three-dimensional structure [1].

Several studies, conducted on these proteins lacking a defined structure, demonstrated that the intrinsic physiological disorder allows the protein to act like a ligand that folds, partially or totally, upon interaction with its targets [2]. These observations gave rise to the concept that partial or total intrinsic disorder, present in many proteins, might have biological significance. The way these proteins fold is part of its function, being an important factor in the interaction with its specific targets. Thus, the function of intrinsically disordered proteins is considered to be directly linked to their folding ability during their interaction with the targets [3,4].

Since intrinsic disorder (ID) is essential for the biological function of various proteins, and might occur in varied environments, the amino acid sequences constituting such disordered regions are specifically determined by the characteristics of their local and global environment. Thus, based on the association of the heterogeneity of ID in proteins with several environmental factors, it is possible to assume that ID might occur in a specific way for each disordered protein (or family of disordered proteins) [3,5].

Comprehensive analysis of several known proteomes has shown that viral proteins have a greater amount of ID compared to human proteins. The content of disordered regions in viral proteins is directly linked to the pathogenicity and oncogenicity of the virus [6]. The presence of large ID regions in viral proteins has several functional implications; some of these regions are indispensable for the functioning of these proteins, for example in the invasion of host cell pathways, as a means of adaptation, accommodation of the virus in hostile habitats, and also to help the virus in the proper management of its genetic material [5,6,7].

The impact of human papillomavirus (HPV) on health was the main motivation for the extensive study and documentation of its characteristic features. The HPVs have more than 150 different genotypes that were fully sequenced and numbered by their order of discovery [8,9].

Currently, HPV is one of the most common sexually transmitted infections, besides being the cause of a series of neoplasms, including cervical, vulvar, vaginal, penile, anal, and head-and-neck cancers. In addition, it is a common cause of genital warts and recurrent respiratory papillomatosis that is somewhat uncommon, yet very severe and etiologically associated with HPV [8,10,11,12].

There are approximately 40 types of HPV that cause infections. The different types are divided into two groups namely High- and Low-Risk, based on their oncogenic potential. According to reports by the International Agency for Research on Cancer and the World Health Organization, HPVs of high oncogenic risk in humans are: types 16, 18, 31, 33, 35, 39, 45, 51, 52, 56, 58, 59, and 66 due to their high prevalence in different types of cancer samples [13,14]. HPV types 6, 11, 40, 42, 43, 44, and 54 are considered to be of low oncogenic risk, causing particularly benign lesions, such as genital warts, low-grade intraepithelial squamous lesions, and laryngeal papillomatosis [13,14,15].

The HPV viral genome encodes nonstructural proteins E1, E2, E3, E4, E5, E6, and E7, required for viral replication and transcription [8,14]. The E6 and E7 proteins are considered to be of high oncogenic risk because of their role in inhibiting cell differentiation. These proteins are associated with the modification of normal epithelial differentiation, blockade of apoptosis, DNA synthesis, and inhibition of cell cycle control [8,16,17]. Therefore, these proteins have been important molecular targets in the search for treatments against HPV.

Studies with E7 proteins from different HPV types have shown a relationship between the different patterns of intrinsic disorder and the degree of viral oncogenicity. E7 proteins from high-risk HPVs, type 16 and 18, show an increase in the size of intrinsically disordered regions relative to that in the low-risk HPV proteins [18]. Other studies propose that intrinsic disorder is relatively abundant even among several established cancer-related proteins [19,20].

The study of intrinsic disorder and its impact on three-dimensional structures of E6 proteins of high and low-risk types of HPV can provide further information about the structural behavior and function of these proteins.

## 2. Results

### 2.1. Analysis of Intrinsic Disorder

Based on data published by the International Agency for Research on Cancer and the World Health Organization [13,14], 15 types of high-risk HPVs most frequently related to cancer, and 12 types of low-risk HPVs most frequently related to benign lesions, were chosen. A type of HPV, related to neither cancer nor benign lesions [13], was chosen as a control in the analyses (Table 1).

The primary sequences of high-risk, low-risk, and control HPV E6 proteins were obtained from the UniProt database (http://www.uniprot.org/).

All sequences were analyzed individually and the results obtained were compared to each other.

Table 1 shows the number of disordered amino acids at the two termini and the overall percentage of intrinsic disorder of E6 proteins, analyzed by the PONDR-FIT software [21].

The N-terminal and C-terminal ends of the high-risk HPV E6 proteins were observed to have a higher number of disordered amino acids and a higher percentage of ID relative to the total length of the sequence when compared to those of the low-risk and control HPV E6 proteins.

**Figure 1:**
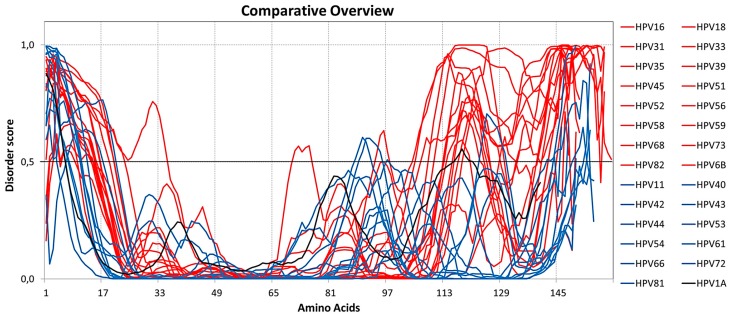
Intrinsic disorder analysis of E6 proteins of high and low-risk human papillomavirus (HPVs), oncogenic and control. The dashed lines at 0.5 of *y*-axis are threshold lines for disordered/structured residues. Residues with a score above this line are predicted disordered, and residues with a score below 0.5 are predicted to be ordered. Intrinsic disorder of HPV E6 proteins of high-risk (red), low-risk (blue), and control (black) are shown.

Analysis of ID distribution shows that the disordered amino acids are predominantly located at the C-terminus of the high-risk HPV E6 proteins, which has a higher amount of ID compared to the low-risk HPV E6. The general comparison between the disordered residues of high and low-risk HPV E6, together with those of the control, reveals the difference in the proportion of ID between the proteins analyzed (Figure 1).

These results show that high-risk HPV E6 proteins present a greater amount of disordered amino acids at their N- and C-termini, suggesting the amount of ID as a possible factor causing the increased oncogenicity related to E6 proteins. Nominé et al. [22] showed that the E6 proteins of various types of HPV present a great variability in the length and sequence of the N-terminal and C-terminal segments. Combining the sequence variability, previously described, with the N-terminal and C-terminal ID distribution pattern found in the present study, it is possible to infer that this combination is one of the determining factors for the varying degrees of oncogenicity of E6 proteins.

To understand the impact of ID on proteins better, it is necessary to analyze the tertiary structure of the regions corresponding to the disordered amino acids, especially at the termini. Till date, the vast majority of the three-dimensional structures of the known E6 proteins are not completely resolved, especially the terminal regions that are still incomplete [23,24]. Therefore, complete tertiary structures of the E6 proteins of high-risk HPV16 and HPV18, low-risk HPV6B and HPV11, and HPV1A control were modeled, so that the ID present at the termini could be analyzed in full.

### 2.2. Molecular Modeling of E6

**Figure 2:**
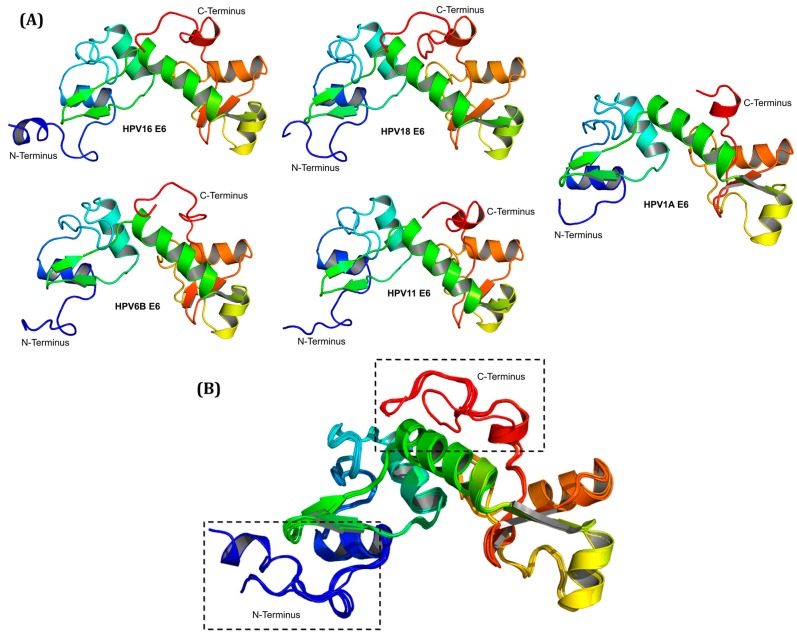
Representation of the three-dimensional structures of E6 proteins. (**A**) Complete structures of the E6 proteins of the HPV type 16 and 18 of high-risk, types 6B and 11 of low-risk, and type 1A control; (**B**) Alignment of the three-dimensional structures of HPV16, HPV18, HPV6B, HPV11, and HPV1A E6 proteins, clearly showing that the major structural differences are localized at the N-terminal and C-terminal ends (black dotted rectangles).

Figure 2A shows the complete three-dimensional structures of E6 proteins of the high-risk types HPV16 and HPV18, low-risk types HPV6B and HPV11, and of HPV1A control (available in supplementary material). Alignment of the structures showed that the major differences are located at the N-terminal and C-terminal ends of the proteins (Figure 2B). The differences observed at both ends of the modeled E6 proteins correspond to the variations found in the length and sequence of these proteins.

### 2.3. Simulation of Molecular Dynamics for RMSF Analysis

**Figure 3:**
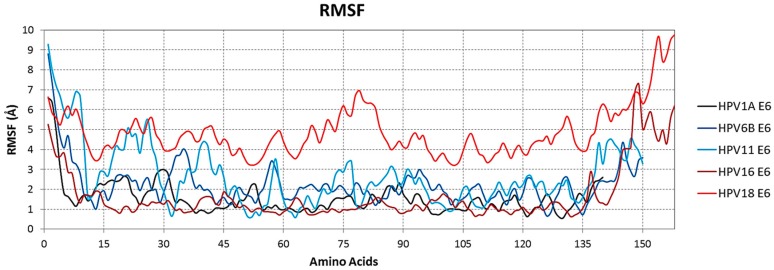
Structural variation of the disordered regions at the N- and C-termini of the proteins analyzed. Root mean square fluctuations (RMSF) values of Cα atom coordinates during the simulation have been plotted to show the structural variation of the N- and C-termini of E6 proteins from HPV16, HPV18, HPV6B, HPV11, and HPV1A. It is observed that high-risk HPVs present greater structural variation at the C-terminal end. All graphs were generated using the Gnuplot 4.6 software [25].

The root mean square fluctuations (RMSF) values for the coordinates of the Cα atoms in the protein structure were obtained over the simulation time using their initial structure as reference to identify the amino acids that provide variable protein characteristics. The results of the RMSF analysis showed that a large number of structural variation occurs in the N- and C-termini of all the E6 proteins modeled. However, the C-terminus of the E6 proteins of the high-risk types 16 and 18 presented greater structural variation in relation to the other E6 proteins (Figure 3), which is consistent with the results obtained using the PONDR-FIT software that showed the intrinsic disorder at the two termini of all proteins analyzed.

For a more detailed comparison of the N- and C-terminal intrinsic disorder between the high- and low-risk E6, the proteins from high-risk HPV type 16 and 18 were chosen because of their direct link to cervical cancer [23,26], E6 of low-risk types 6B and 11 were chosen because of their high prevalence in benign lesions related to HPV [26], and E6 of type 1A was chosen due to its lack of relation with either cancer or benign lesions [13].

**Figure 4:**
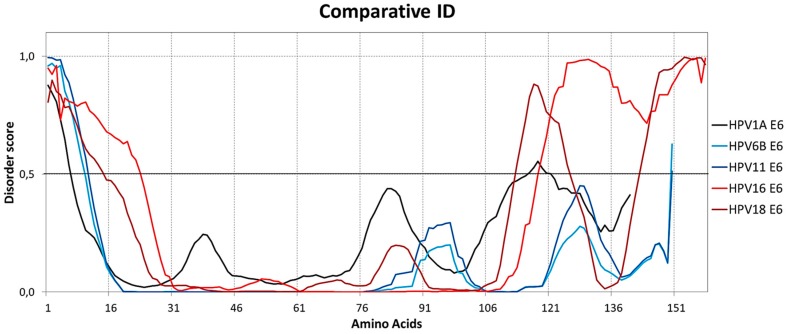
Comparison of intrinsic disorder between E6 of HPV16 and HPV18 (high-risk), HPV6B and HPV11 (low-risk), and HPV1A (control). High-risk HPV16 and HPV18 E6 proteins have considerably longer regions of intrinsic disorder at both ends, especially at the C-terminus. The dashed lines at 0.5 of *y*-axis are threshold lines for disordered/structured residues. Residues with a score above this line are predicted disordered, and residues with a score below 0.5 are predicted to be ordered. These results were obtained using the PONDR-FIT tools [21].

Comparison of the two high-risk E6 proteins (HPV16 and HPV18) with the two low-risk E6 proteins (HPV6B and HPV11) shows a large difference in the amount of ID at the two termini, with E6 of HPV16 and HPV18 presenting 39.9% and 27.2% of disordered amino acids respectively, E6 of HPV6B and HPV11 presenting 6.7% and 7.3%, respectively and E6 of HPV1A presenting 8.6%, thereby showing that the amount of ID is significantly higher in both E6 termini of HPV16 and HPV18 (Figure 4).

**Figure 5:**
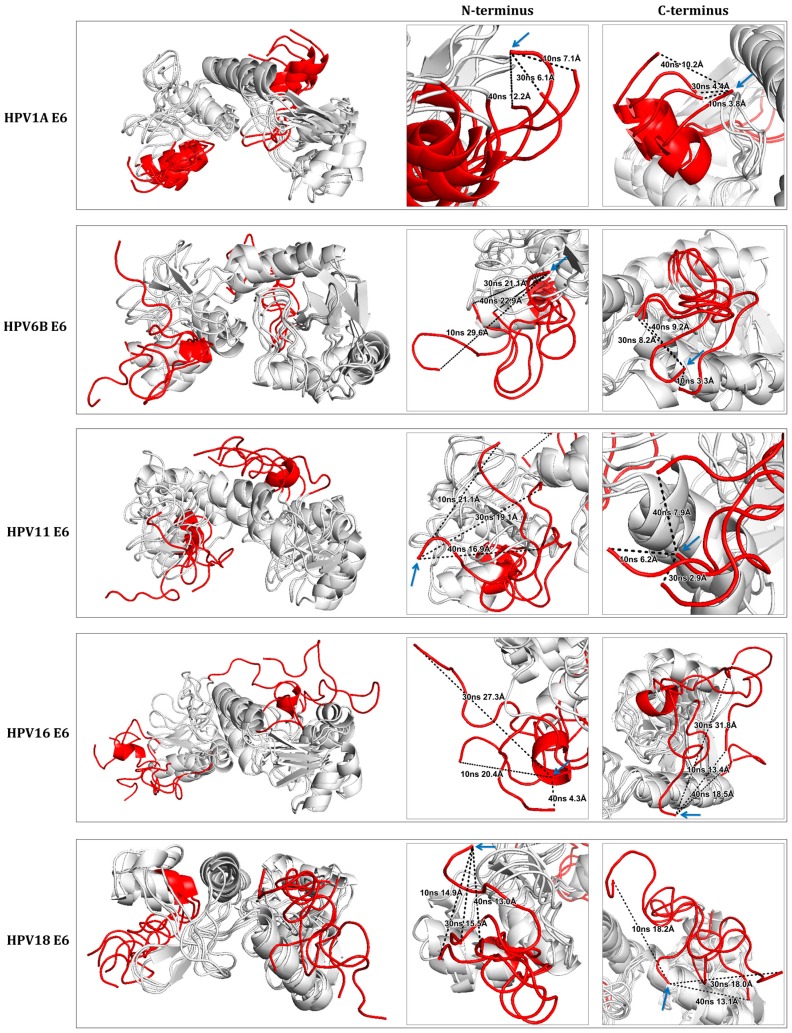
Comparison between the different structural conformations of E6 proteins from HPV16, HPV18, HPV6B, HPV11, and HPV1A by molecular dynamics simulation. The N- and C-termini of the structures are highlighted in red and the central region between the terminals is shown in gray. The panels, next to the aligned structures, highlight the two termini with the black dotted lines showing the distance of the variation of the same amino acids at times 0 (indicated by the blue arrow), 10, 30, and 40 ns. It is observed that the structural variation occurs mainly in the terminal regions of all the structures. Distances were measured using PyMOL 2.0 software [27]. Dotted line: the distance of the variation of the same amino acids.

Alignment of the three-dimensional structures, obtained during molecular dynamics simulation, was done using PyMOL 2.0 software [27]. Four conformations of each E6 protein of HPV16, HPV18, HPV6B, HPV11, and HPV1A, corresponding to times 0, 10, 30, and 40 ns (Figure 5) were aligned. As expected, the N- and C-terminal regions, upon visual inspection, were found to be structurally most variable.

The mean of structural variation, calculated based on the distances of the conformations at 0, 10, 30, and 40 ns, using the first amino acid of the N-terminal and the last amino acid of C-terminal end of E6 from HPV1A, HPV6B, HPV11, HPV16, and HPV18 was 8.63 Å, 24.53 Å, 19.03 Å, 17.33 Å, and 14.47 Å, respectively, at the N-terminal and, 6.13 Å, 6.90 Å, 5.67 Å, 21.23 Å, and 16.43 Å, respectively, at the C-terminal end.

The structure of the non-cancer-related HPV1A (control) E6 was the only one that showed lower structural variation at both its termini. The low-risk HPV6B and HPV11 E6 structures showed an increase in the structural variation at the termini compared to that in the control E6, primarily in the N-terminus that showed greater variation even when compared to the high-risk HPV16 and HPV18 E6. The structures of high-risk HPV16 and HPV18 E6 presented structural variation in both termini, though to a greater extent in the C-terminus compared to the other proteins. Despite the large structural variation of both termini of the E6 proteins analyzed, the central regions of the structures were observed to have little variation relative to the termini (Figure 5).

### 2.4. Analysis of Electrostatic Potential and Hydrophobicity Profile

The distribution of electrostatic potential and surface hydrophobicity of the E6 proteins were obtained through the ChimeraX software [28].

**Figure 6:**
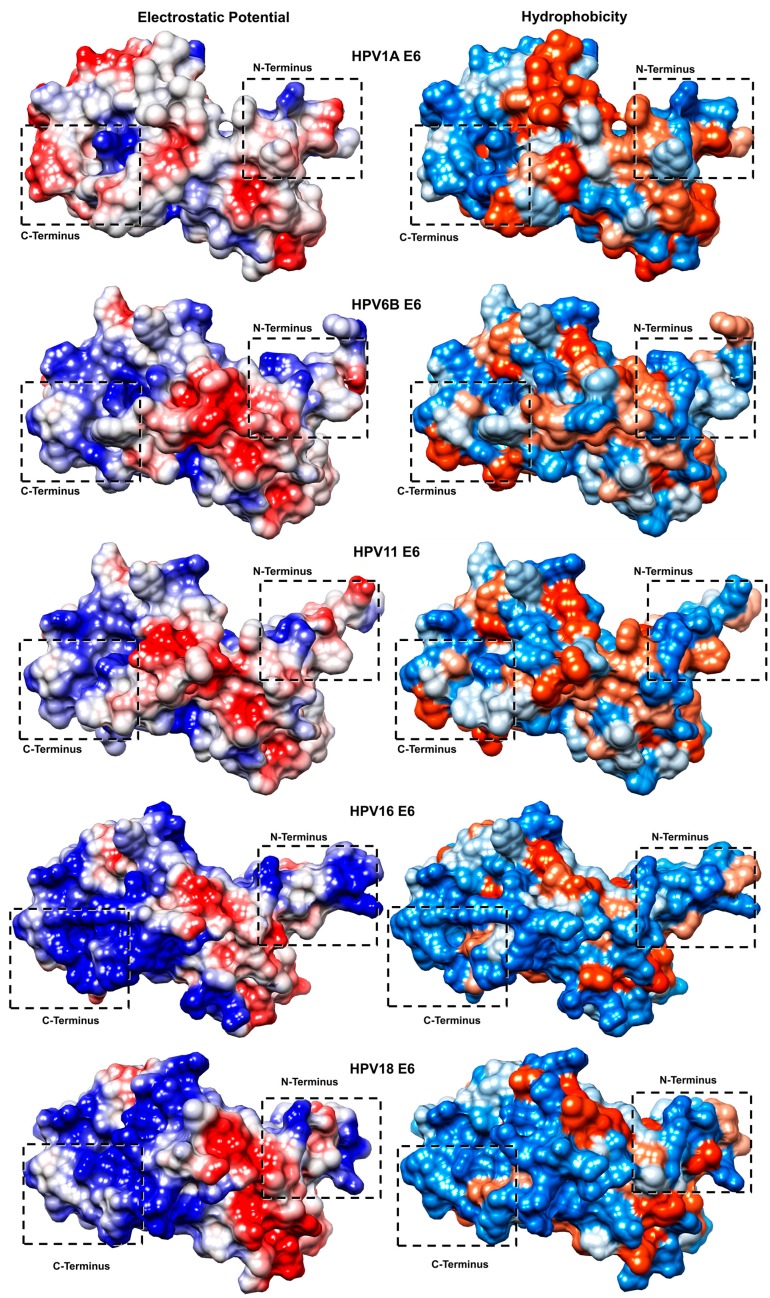
Distribution of electrostatic potential and surface hydrophobicity. The electrostatic potential distribution is shown in blue in the positively charged regions, red in the negatively charged regions, and white in the neutral regions. The hydrophobicity distribution on the protein surfaces is shown in blue for hydrophilic regions, orange for hydrophobic regions, and blank for neutral regions. The rectangles dotted in black show the regions corresponding to the N- and C-terminal surfaces. HPV1A, HPV6B, and HPV11 (control and low-risk) oncogenic E6 proteins have a greater proportion of neutral and hydrophobic electrostatic characteristics in the regions corresponding to the N- and C-terminal surfaces. The distribution of electrostatic potential and hydrophobicity of the surfaces of highly oncogenic HPV16 and HPV18 E6 proteins present predominantly positive and hydrophilic characteristics at the termini of both structures. The rectangles dotted in black show the regions corresponding to the N- and C-terminal surfaces.

For visual analysis, the structures were all equally positioned, with the C-terminus at the left and N-terminus at the right end of all the images contained in Figure 6.

Surface analyses showed a similar pattern in the distribution of electrostatic potential and hydrophobicity between the high-risk HPV16 and HPV18 E6 structures. The same was true for the low-risk HPV6B and HPV11 E6 structures that share a pattern similar to each other as well as to that of HPV1A E6 (control) (Figure 6).

Positively charged and hydrophilic regions were predominant at the C-terminus of all the structures, with the largest regions observed in high-risk HPV16 and HPV18 E6 proteins. The electrostatic surface of the low-risk HPV6 and HPV11 E6 and HPV1A E6 structures showed a predominance of neutral and hydrophobic regions compared to that in the high-risk E6 structures.

The propensity of intrinsic disorder in proteins is related to the physicochemical characteristics of the amino acids that compose them. Studies have shown that hydrophobic residues are rarely encountered in most regions of intrinsic disorder, whereas hydrophilic and positively charged residues are found in abundance [29,30]. These specific characteristics of predominance of positively charged and hydrophilic regions at the N- and C-termini were found to correspond to the higher ID regions of the high-risk oncogenic E6 proteins.

## 3. Discussion

The presence of intrinsically disordered regions in proteins is directly related to signaling and regulation of the cell cycle [3]. In previous studies, intrinsic disorder has been linked to different types of cancer-related proteins [18,20], such as the p53 protein [31], P57 kip2 [32], Bcl-x L and Bcl-2 [33], C-Fos [34], TC-1 [35], and EWS [36].

Viruses are obligate intracellular parasites, because their genomes are not large enough to encode all the functions necessary to reproduce their progeny independently. Thus, viruses are categorically dependent on host-cell functions [37]. ID, commonly found in viral proteins, allows plasticity in its interaction with different targets and also promotes its adaptation to various environmental conditions [38].

ID also confers high mutation rates on viral proteins, giving rise to new viral types, subtypes, and variants [37,38]. A recent study has shown that viral variants of the HPV16 E6 protein show conservation of the disordered regions, implicating a significant function of these proteins (results not yet published by our group).

To date, there are several partial structures of the E6 proteins that has been solved experimentally, the structure of HPV16 E6 is the best studied and described [23,24]. The complete structural determination of E6 proteins of the high-risk types 16 and 18, low-risk types 6B and 11, and control 1A, in this study, will help to analyze the structural behavior of the intrinsically disordered regions along the protein length, as well as to understand the characteristics of electrostatic potential and hydrophobicity profile of the surface of these proteins.

RMSF analysis showed that, during the molecular dynamics simulation, both ends, mainly the C-terminus of the high-risk E6 proteins, present greater structural variation, corroborating the results presented by the PONDR-FIT tool. The analyses of electrostatic potential and hydrophobicity profile also confirmed the prediction of ID and structural variability, showing that the surface regions at the termini present prominent positively charged and hydrophilic characteristics, corresponding to the established physicochemical characteristics of regions with IDs, already described in other proteins [29,30].

Although the best-described function of E6 is to induce degradation of p53, several studies have indicated E6 proteins to have many other molecular targets. One of the major binding sites described for E6 protein is located in its C-terminal domain, the PDZ (postsynaptic density 95/disc large/zonula occludens-1) binding motif [24,39]. The PDZ binding motif is specifically conserved among the high-risk HPV E6 proteins and is essential for recognizing, binding, and enhancing the degradation of various PDZ domain-containing proteins, such as discs large homolog 1 (DLG1), discs large homolog 4 (DLG4), SCRIB (scribble homolog), membrane-associated guanylate kinase inverted 1 (MAGI1), and tyrosine-protein phosphatase non-receptor type 13 (PTPN13) [39,40]. Several studies suggest that the motif of binding to the PDZ domain is particularly important for cell transformation and tumorigenesis [39,41]. The recent study by Yoshimatsu et al. [39] shows that the PDZ domain-binding motif located at the C-terminus of HPV type 16 E6 is critical to induce proliferation, anchorage-independent growth, and tumorigenic cellular potential of the virus. These data strengthen the results obtained in the present study, where the highest proportion of intrinsic disorder and structural variation was observed in the C-terminus of the high-risk E6 proteins, indicating that ID can confer a better recognition and interaction capacity with PDZ domains of their molecular targets, suppressing their functions more efficiently, and increasing the tumorigenesis caused by the virus.

Corroborated by the studies conducted by Uversky et al. [19] and Nicolau and Giuliatti [18], where differences in the content of ID were observed in E6 and E7 oncoproteins of high-risk HPV, the results obtained in our study reveal that, the N-terminal and C-terminal amino acids of the high and low-risk E6 proteins are responsible for causing variation in the degree of ID and, consequently, explain the variation of structure and dynamics between the E6 structures. These differences in the structures may implicate a specific behavior of each E6, when complexed with its target proteins, imparting a possible advantage over molecular interaction.

The variation in the amount of ID and structural characteristics might indicate that these factors are responsible for the differences in degree of oncogenicity and viral persistence of the E6 proteins. These data need to be confirmed further using the E6 proteins of high and low-risk HPV, and also in complex with their main targets, such as p53, E6AP, and other possible molecular targets. The acquired knowledge would contribute to studies that seek efficient prophylactic methods and drug therapies against HPV and related pathologies.

## 4. Materials and Methods

### 4.1. Obtaining the Primary Sequences

The types of high and low-risk HPV, used in this study, were chosen based on data published by the International Agency for Research on Cancer and the World Health Organization [13,14].

The primary sequences of the high and low-risk HPV E6 proteins are presented in Table 2. These data were obtained from the UniProt Database (http://www.uniprot.org/).

### 4.2. Prediction and Analysis of Intrinsic Disorder

Currently, there are several computational tools for predicting intrinsic disorder. Based on the different methodologies available, the PONDR-FIT software [21] was used to predict regions of intrinsic disorder in E6 proteins of different HPV types.

PONDR-FIT employs the unification of the methodologies of six predictors namely, PONDR^®^ VLXT, PONDR^®^ VL3, PONDR^®^ VSL2, IUPred, FoldIndex, and TopIDP. The output data are composed of a table that individually scores each of the amino acids in the sequence, indicating the probability of each residue being structured or disordered. While the values less than 0.49 represent structured amino acids, those greater than 0.5 represent the intrinsically disordered amino acids. The PONDR-FIT consensus predictor analyzes each amino acid throughout the sequence, taking into account the characteristics of the neighboring amino acids. The prediction results of the six tools are weighed in a sliding window of 21 residues, centered on the residue being analyzed [21,42].

### 4.3. Macromolecular Modeling and Ab Initio Modeling

Primary structure of the HPV16 E6 protein (P03126) was obtained from the public database UniProt (http://www.uniprot.org). The structure of HPV16 E6 oncoprotein, obtained by X-ray crystallography, in complex with the LXXLL peptide of the E6AP protein (PDB ID: 4GIZ), was used as a template structure [23].

The structure of HPV16 E6 was partly modeled by homology modeling. For this, the server @TOME-2 [43], which uses the computer program MODELLER [44], was used. Five modeled structures were generated and evaluated by ERRAT [45], PROCHECK [46], and visual inspection.

Residues 1-8 and 151-158, in the N- and C-terminal regions of HPV16, respectively, were absent in the template structure and were modeled ab initio. The Cyclic Coordinate Descent (CCD) module [47], implemented in the computer program Rosetta 3.1 [48], was used to generate 1000 templates from HPV16 E6, which were ranked by a score function using the computer programs ModFOLD [49] and QMEAN [50]. The top 20 modeled structures (the 10 highest scores sorted by each scoring function without redundancy) were evaluated by MQAPs and visual inspection. In order to obtain high-quality structures, the side chains (rotamers) of the final structure, were re-modeled using SCWRL4 [51].

The structure of the fully modeled HPV16 E6 protein was used as a template for the homology modeling of the E6 proteins from HPV1A, HPV6B, HPV11, and HPV18. Using the computer program MODELLER, five structures for each target were generated.

The best structure was selected and analyzed by visual inspection and MQAPs. The side chain modeling was performed by the computer program SCWRL4.

### 4.4. Analysis of Electrostatic Potential and Hydrophobicity Profile

For prediction of the electrostatic potential distribution and hydrophobicity profile of the protein surface, UCSF ChimeraX software [28] was used.

UCSF ChimeraX calculates the electrostatic surface potential of proteins according to Coulomb’s Law. For prediction of the hydrophobicity profile, it attributes a property called kdHydrophobicity to the amino acids individually; its values vary according to the hydrophobicity scale of Kyte and Doolittle [52].

### 4.5. Simulation of Molecular Dynamics

For molecular dynamics simulations, we used the computer program NAMD 2.8 [53] with CHARMM27 [54], in which the parameters used in the energy function are implemented for all atoms. For visualization and analysis of the simulation results, we used the graphics program VMD [55].

In each simulation, the structure was solvated in a water shell 20 Å thick. The simulation was performed under normal temperature and pressure (NTP), and the temperature was raised slowly to 310 K in the first 62 ps. The total time of each simulation was 40 ns.

### 4.6. Visualization and Visual Inspection of Models

The obtained models were visualized and inspected through the software UCSF ChimeraX [28] and PyMOL 2.0 [27].

## 5. Conclusions

By means of in silico analysis of primary sequences, molecular modeling, structural analysis, and molecular dynamics analysis, the present study showed that all the high and low-risk oncogenic E6 proteins analyzed have ID regions clustered at their N- and C-termini. However, high-risk HPV E6 proteins exhibit a much larger amount of disorder, especially at the C-terminus. The molecular modeling and simulation of molecular dynamics of the tertiary structures of HPV16 and HPV18 (high-risk), HPV6B and HPV11 (low-risk), and HPV1A (control) E6 proteins allowed the visualization of the structural variation at the termini of the proteins analyzed, evidencing the relationship between degree of oncogenicity and amount of ID at the termini. These results might implicate a direct relationship of the amount of ID to the degree of oncogenicity. These results are of vital importance and should be considered in future studies for the development of drugs based on molecular targets.

## Figures and Tables

**Table 1:** Number of disordered amino acids and general percentage of intrinsic disorder in E6 proteins.

HPV Type	Classification	#Residues	% ID	Disordered Amino Acids N-Terminal	Disordered Amino Acids C-Terminal
HPV1A	Control	140	8.6	6	6
HPV16	High-Risk	158	39.9	23	40
HPV18	High-Risk	158	27.2	14	29
HPV31	High-Risk	149	38.9	20	38
HPV33	High-Risk	149	35.6	16	37
HPV35	High-Risk	149	33.6	13	37
HPV39	High-Risk	158	31.6	8	42
HPV45	High-Risk	158	31.6	13	37
HPV51	High-Risk	151	33.1	18	32
HPV52	High-Risk	148	45.9	35	33
HPV56	High-Risk	155	27.1	20	22
HPV58	High-Risk	149	36.9	18	37
HPV59	High-Risk	160	25.6	15	26
HPV68	High-Risk	158	25.3	11	29
HPV73	High-Risk	148	14.2	14	7
HPV82	High-Risk	151	36.4	13	42
HPV6B	Low-Risk	150	6.7	9	1
HPV11	Low-Risk	150	7.3	10	1
HPV40	Low-Risk	154	3.9	6	0
HPV42	Low-Risk	150	11.3	10	7
HPV43	Low-Risk	155	11.0	7	10
HPV44	Low-Risk	150	7.3	11	0
HPV53	Low-Risk	154	14.3	20	2
HPV54	Low-Risk	144	13.9	12	8
HPV61	Low-Risk	146	4.8	3	4
HPV66	Low-Risk	155	10.3	11	5
HPV72	Low-Risk	148	4.7	6	1
HPV81	Low-Risk	154	7.8	10	2

#Residues number refers to the number of amino acid residues.

**Table 2:** Primary sequences of high and low-risk HPV E6 proteins.

HPV Type	Classification	ID UniProtKB	#Residues
HPV1A	Control	P06929	140
HPV16	High-Risk	P03126	158
HPV18	High-Risk	P06463	158
HPV31	High-Risk	P17386	149
HPV33	High-Risk	P06427	149
HPV35	High-Risk	P27228	149
HPV39	High-Risk	P24835	158
HPV45	High-Risk	P21735	158
HPV51	High-Risk	P26554	151
HPV52	High-Risk	P36814	148
HPV56	High-Risk	P24836	155
HPV58	High-Risk	P26555	149
HPV59	High-Risk	B9UPD9	160
HPV68	High-Risk	Q7KYK8	158
HPV73	High-Risk	Q82005	148
HPV82	High-Risk	Q9IR59	151
HPV6B	Low-Risk	P06462	150
HPV11	Low-Risk	P04019	150
HPV40	Low-Risk	P36812	154
HPV42	Low-Risk	P27229	150
HPV43	Low-Risk	P19709	155
HPV44	Low-Risk	P19710	150
HPV53	Low-Risk	Q17UC4	154
HPV54	Low-Risk	Q81018	144
HPV61	Low-Risk	Q80948	146
HPV66	Low-Risk	Q80955	155
HPV72	Low-Risk	Q81997	148
HPV81	Low-Risk	Q705E9	154

#Residues number refers to the number of amino acid residues.

## Supplementary Materials

Supplementary materials can be found at www.mdpi.com/1422-0067/19/1/198/s1.

## Abbreviations

HPV	Human Papillomavirus
ID	Intrinsic Disorder
RMSF	Root Mean Square Fluctuations
Cα	Alpha Carbon
